# Quantification of choroidal hyperreflective layer: A swept-source optical coherence tomography study

**DOI:** 10.1371/journal.pone.0294476

**Published:** 2023-11-29

**Authors:** So Min Ahn, Myung-Sun Song, Ariunaa Togloom, Jaeryung Oh

**Affiliations:** Department of Ophthalmology, Korea University College of Medicine, Seoul, South Korea; Hangil Eye Hospital / Catholic Kwandong University College of Medicine, REPUBLIC OF KOREA

## Abstract

**Purpose:**

To investigate variation in reflectivity of choroidal layers in normal eyes.

**Methods:**

From the swept-source optical coherence tomography database, we retrospectively included eyes with a normal fundus. Choroidal reflectivity was measured on the horizontal and vertical B-scan optical coherence tomography images. The optical barrier of the choroid was defined as the first hill in the middle of the reflectance graph from the retinal pigment epithelium-Bruch’s membrane complex to the chorioscleral junction.

**Results:**

The optical barrier of the choroid was identified in 91 eyes of 91 individuals. The amplitude of peak reflectivity of the optical barrier of the choroid at macular center (142.85 ± 15.04) was greater than those in superior (136.12 ± 14.08) or inferior macula (135.30 ± 16.13) (P = 0.028, P = 0.008, respectively). Latency between the peak of the retinal pigment epithelium-Bruch’s membrane complex and the optical barrier of the choroid at macular center (48.11 ± 13.78 μm) was shorter than those in nasal macula (55.58 ± 19.21 μm) (P = 0.021). The amplitude of the peak reflectivity of the optical barrier of the choroid in the center negatively correlated with the latency between the retinal pigment epithelium-Bruch’s membrane complex and the optical barrier of the choroid (P < 0.001).

**Conclusion:**

An optical barrier exists in the inner choroid of the normal eye. Its depth depends on the location within the macula. Further studies are mandatory to evaluate variations in the barrier in the eyes with chorioretinal disease.

## Introduction

Since optical coherence tomography (OCT) was first used for fundus imaging in 1991, its resolution has improved [1–3]. The retinal and choroidal tissue layers can be identified by the difference in reflectivity on OCT [1]. A greater refractive difference at the boundary between different tissues or higher scattering in the tissue layer correlated with higher reflectivity [4]. In the retina, repetition of hyperreflective and hyporeflective layers was detected using OCT. The retinal layers that show a hyperreflective band on OCT are the nerve fiber layer, inner plexiform layer, outer plexiform layer, external limiting membrane, ellipsoid zone of the photoreceptors, cone interdigitation with the retinal pigment epithelium (RPE), and RPE-Bruch’s membrane (RPE-BM) complex [1]. The retinal layers that show a hyporeflective band on OCT are the ganglion cell layer, inner nuclear layer, outer nuclear layer, myoid zone of the photoreceptors, and outer segments of the photoreceptors [1].

In addition to those for measuring the retina, various methods for measuring the choroid using OCT devices have gradually been developed [5, 6]. Previous studies have reported an association between the choroidal thickness, choroidal vascular index (CVI), and chorioretinal disorders [7–10]. Although these studies using OCT reflectivity in the choroid have provided information about changes in the vascular components of the choroid, few studies have presented detailed changes in choroidal components other than the vessels [7, 8, 11, 12]. However, the choroid comprises stroma and blood vessels of different sizes, and varying choroidal reflectivity is shown on OCT [1]. Abundant melanocytes are located in the choroidal stroma, and the absorption and reflectance of light in melanocytes leads to reflectivity on OCT [12, 13]. These variations in reflectivity from the choroidal stroma may act as an optical barrier to prevent light reflection.

In this study, we hypothesized that OCT devices could reflect the optical properties of the choroid. In normal fundus eyes, we obtained plots of choroidal reflectivity using swept-source OCT (SS-OCT) and characterized the pattern of the plot.

## Materials and methods

This retrospective study was approved by the Institutional Review Board of the Korea University Hospital, Seoul, Korea. Medical Center waived the need to obtain informed consent from the participants. The study adhered to the tenets of the Declaration of Helsinki. We included OCT images with a normal fundus from the OCT database at Korea University Hospital. After reviewing the medical records of individuals with normal eyes, we excluded the images of enrolled eyes with chorioretinal diseases and those of low image quality. Each individual underwent comprehensive ophthalmic examination, including OCT and biometry, between May 2016 and June 2022. Axial length was measured using IOLMaster (version 5.4, Carl Zeiss Meditec, Germany).

### Optical coherence tomography

In this study we used SS-OCT. DRI OCT Triton (Topcon Corp., Tokyo, Japan) was used to obtain the OCT images. The OCT device has a central wavelength of 1,050 nm, a speed of 100,000 A-scans/s, a horizontal resolution of 20 μm, and an axial resolution of 8 μm [7]. This device contained a camera to obtain color images of the fundus. Five 9-mm horizontal and five vertical line raster scan protocols centered on the fovea were used for each individual and averaged from multiple B-scans to improve the signal-to-noise ratio. The choroidal thickness was measured in the subfoveal area on a horizontal B-scan image centered on the fovea using a previously presented method [7]. The CVI, choroidal total area, luminal area, and stromal area with a width of 1,500 μm centered on the fovea were measured using a previously presented method [7]. The choroidal area was manually selected using a polygon tool, and image binarization of the selected image was performed using an auto local thresholding tool with the Niblack method [7].

### Measurements of choroidal reflectivity

**Fig 1:**
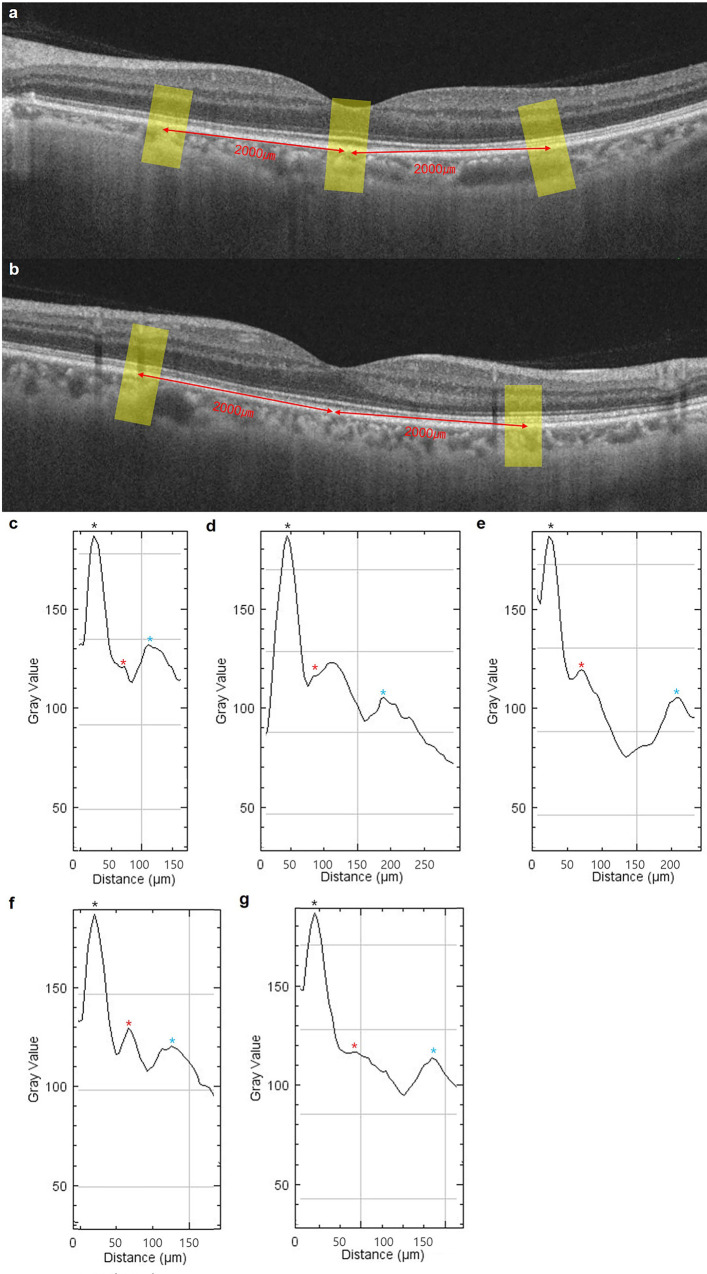
Measurement of choroidal reflectivity on OCT. Choroidal reflectivity was measured in horizontal (a) and vertical (b) B-scan images. A 500 μm-wide linear line was drawn perpendicular to Bruch’s membrane at the center of the fovea, and nasal, temporal, superior, and inferior locations from the fovea center (yellow lines in a and b). Gray values according to distance were analyzed at each location (c. nasal, d. center, e. temporal, f. superior, and g. inferior). The black asterisk indicates RPE-BM complex reflectivity, the red asterisk indicates peak reflectivity of the optical barrier of the choroid (OBC), and the blue asterisk indicates the chorioscleral interface.

Choroidal reflectivity was measured on horizontal and vertical B-scan OCT images centered on the fovea using ImageJ software (http://imagej.nih.gov/ij/; provided in the public domain by the National Institutes of Health) in the macular center, nasal, temporal, superior, and inferior macular areas. A horizontal B-scan image centered on the fovea was selected for measuring the reflectivity in the macular center, nasal, and temporal areas. A vertical B-scan image centered on the fovea was selected for measuring the reflectivity in the superior and inferior macular areas. After uploading the B-scan images using the ImageJ software, the images were changed to 8-bit images. The image scale was changed from pixel size to length (μm) by “Set scale”. Using the line tool of ImageJ, a 500-μm width-linear line was drawn perpendicular to RPE-BM complex and covering all layers of the retina and choroid at the center of the fovea and four (nasal, temporal, superior, and inferior) locations 2,000 μm away from the foveal center (Fig 1A and 1B). After drawing the line, a “plot profile” was applied to obtain the gray value according to the distance (Fig 1C–1G). The RPE-BM complex reflectivity was determined as the highest gray value in the area corresponding to the RPE-BM complex layer. The mean choroidal reflectivity was defined as the mean reflectivity between the RPE-BM complex layer (reflectivity after peak reflectivity) and chorioscleral interface.

The OBC was defined as a hill appearing in the middle of the reflectance graph from the RPE-BM complex to the chorioscleral junction (Fig 1C–1G). If several hills appeared, the first hill was determined to be the OBC. The amplitude of the peak reflectivity in the OBC and RPE-BM was measured. The latency between the RPE-BM complex and OBC peaks was measured. If the plot slope continuously decreased without an OBC within the plot distance, the NaN (not a number) was determined.

### Variations in the optical barrier of the choroid in the diseased eyes

For comparison, we included several eyes with chorioretinal diseases. And we measured choroidal reflectivity in the same way as when measuring the choroid of normal subjects.

### Statistical analyses

Statistical analyses were performed using SPSS software (version 20.0; IBM, Armonk, NY, USA). Pearson’s correlation was used to analyze the relationship between continuous variables. In cases of multiple comparisons, Analysis of Variance (ANOVA) test was used with Bonferroni post hoc test. Statistical significance was defined as P < 0.05.

## Results

Overall, 91 eyes of 91 individuals with a normal fundus were included; 11 (12.1%) eyes of 91 individuals had high myopia without pathologic myopic degeneration. The mean age of the individuals was 51.41 ± 18.26 years, and the mean subfoveal choroidal thickness was 253.33 ± 64.08 μm (Table 1).

**Table 1:** Characteristics of the study participants.

Variables	N = 91
Male (%)	46 (50.5%)
Age, years (range)	51.41±18.26 (21–87)
OD/OS	58/33
HTN (%)	18 (19.8%)
DM (%)	17 (18.7%)
AL, mm	24.71±1.49 (21.78–28.64)
Spherical equivalent	-0.69±2.44 (-8.50 - +3.50)
High myopia^a^ (%)	11 (12.1%)
SFCT, μm	253.33±64.08 (168–327)
CVI, %	61.86±7.76 (51.11–74.01)
TCA, μm^2^	581,126±296,403
LA, μm^2^	369,273±211,406
SA, μm^2^	211,853±94,317

HTN, Hypertension; DM, Diabetic Mellitus; AL, Axial length; SFCT, Subfoveal choroidal thickness; CVI, Choroid vascular index; TCA, Total choroidal area; LA, Luminal area; SA, Stromal area

^a^ High myopia was defined as an axial length of > 26.0 mm or a spherical equivalent value of ≤ -6.00 D.

### Distribution of the optical barrier of the choroid in the normal eyes

The OBC was identified in 98.9%, 97.8%, 100%, 100%, 98.9% in the center, nasal, temporal, superior, and inferior areas, respectively (Table 2), with no significant differences between the locations (P = 0.478). The mean peak amplitude of the OBC was 142.85 ± 15.04, 138.76 ± 14.20, 141.27 ± 14.33, 136.12 ± 14.08, and 135.30 ± 16.13 in the center, nasal, temporal, superior and inferior areas, respectively, with significant differences between the locations (P = 0.003) (Table 2). In particular, the peak amplitude of the OBC was higher in the center than in the superior and inferior areas (P = 0.028 and P = 0.008, respectively) (Table 3). The latency between the RPE-BM peak complex and OBC peaks was 48.11 ± 13.78, 55.58 ± 19.21, 50.24 ± 14.29, 52.91 ± 15.92, and 52.46 ± 16.29 μm in the center, nasal, temporal, superior and inferior areas, respectively (Table 2). The latency between the RPE-BM peak and OBC peaks in the nasal area was significantly longer than that in the center (P = 0.021) (Table 3).

**Table 2:** Characteristics of reflectivity in the choroid by location.

	Center	Nasal	Temporal	Superior	Inferior	P- value^a^
Presence of the OBC, number of eyes	90 (98.9%)	89 (97.8%)	91 (100%)	91 (100%)	90 (98.9%)	0.478
Peak reflectivity of the OBC	142.85±15.04	138.76±14.20	141.27±14.33	136.12±14.08	135.30±16.13	0.003
Peak reflectivity of the RPE-BM complex	206.99±9.55	203.11±9.41	202.35±9.43	204.82±12.21	199.26±9.38	<0.001
Latency from peak reflectivity between the RPE-BM complex to the OBC	48.11±13.78	55.58±19.21	50.24±14.29	52.91±15.92	52.46±16.29	0.029
Mean choroidal reflectivity	114.16±16.56	123.37±19.18	115.52±16.52	112.35±15.73	112.34±19.19	<0.001

OBC, Optical barrier of the choroid; RPE, Retinal pigment epithelium; BM, Bruch’s membrane

^a^ ANOVA test, P<0.05 was considered to be statistically significant.

**Table 3:** Post-hoc analysis of the characteristics of reflectivity (Bonferroni).

	Peak reflectivity of OBC	Peak reflectivity of RPE-BM complex	Latency from peak reflectivity between RPE-BM to OBC	Mean choroidal reflectivity
Center vs. Nasal	0.689	0.112	0.021	0.006
Center vs. Temporal	1.000	0.025	1.000	1.000
Center vs. Superior	0.028	1.000	0.600	1.000
Center vs. Inferior	0.008	< 0.001	0.779	1.000
Nasal vs. Temporal	1.000	1.000	0.254	0.032
Nasal vs. Superior	1.000	1.000	1.000	< 0.001
Nasal vs. Inferior	1.000	0.119	1.000	< 0.001
Temporal vs. Superior	0.221	1.000	1.000	1.000
Temporal vs. Inferior	0.080	0.431	1.000	1.000
Superior vs. Inferior	1.000	0.003	1.000	1.000

OBC, Optical barrier of the choroid; RPE, Retinal pigment epithelium; BM, Bruch’s membrane

The average number of observed reflectance peaks between the PRE-BM complex and the chorioscleral junction was 5.76 in the center, 3.88 in the nasal, 4.78 in the temporal, 5.56 in the inferior, and 6.37 in the superior macular areas. Among the participants, 55 exhibited two or more reflectance peaks, and 36 displayed one or zero reflectance peaks between the PRE-BM complex and chorioscleral junction. Patients with one or zero reflectance peaks tended to be older and had less luminal area than those with two or more reflectance peaks (Table 4).

**Table 4:** Factors influencing the number of reflectance peaks between the RPE-BM complex and chorioscleral junction.

	Two or more peaks (n = 55)	One or zero peaks (n = 36)	P-value*	Univariate		Multivariate	
				P-value†	OR (95% CI)	P-value†	OR (95% CI)	
Age	46.49±16.45	58.92±18.55	0.002	0.002	1.041(1.015–1.068)	0.010	1.048(1.012–1.087)	
AL	24.63±1.43	24.85±1.64	0.612	0.591	1.102(0.774–1.567)			
SFCT	289.96±74.85	219.78±75.36	<0.001	<0.001	0.987(0.980–0.994)	0.236	0.994(0.983–1.004)	
CVI	66.03±4.29	65.92±6.90	0.928	0.919	0.996(0.922–1.076)			
TCA	695,829.90±236,539.88	494,795.50±145,053.90	<0.001	<0.001	1.000(1.000–1.000)	0.007	1.000(1.000–1.000)	
LA	461,541.60±161,989.90	330,291.68±112,728.05	<0.001	<0.001	1.000(1.000–1.000)	0.020	1.000(1.000–1.000)	
SA	234,288.30±80,060.14	164,503.82±45,351.95	<0.001	<0.001	1.000(1.000–1.000)			
Peak reflectivity of the RPE-BM complex at macular center	206.20±10.32	207.55±7.90	0.484	0.503	1.016(0.970–1.063)			
Mean choroidal reflectivity at macular center	110.34±15.07	120.65±17.27	0.005	0.005	1.041(1.012–1.070)	0.976	1.001(0.955–1.048)	
Peak reflectivity of the OBC at macular center	143.22±16.70	141.49±12.22	0.571	0.0593	0.992(0.965–1.021)			
Latency from peak reflectivity between the RPE-BM complex to the OBC at macular center	96.57±24.82	95.67±31.75	0.887	0.879	0.999(0.983–1.014)			
Ratio of reflectivity of the OBC to the RPE-BM complex at macular center	69.52±7.90	68.21±6.18	0.381	0.403	0.975(0.919–1.034)			

*Independent t-test

†Binary logistic regression analysis

### Factors associated with the peak reflectance and the latency of the optical barrier of the choroid in the normal eyes

The mean peak reflectivity of the OBC did not correlate with age (*r* = 0.184, P = 0.081). However, at the macular center, the peak reflectivity of the OBC correlated with CVI and RPE-BM complex reflectivity (*r* = -0.222, P = 0.035; *r* = 0.216, P = 0.041, respectively) (Table 5). The amplitude of the peak reflectivity of the OBC negatively correlated with the latency between RPE-BM complex reflectance and OBC (*r* = -0.435, P < 0.001) (Table 5).

**Table 5:** Correlation between reflectivity in the choroid and other parameters at the macular center.

	Peak reflectivity of the OBC	Ratio of reflectivity of the OBC to RPE-BM complex	Latency from the RPE-BM complex to OBC	Mean choroidal reflectivity
Variables	*r* (p-value^a^)	*r* (p-value^a^)	*r* (p-value^a^)	*r* (p-value^a^)
Age	0.167 (0.116)	0.158 (0.137)	-0.014 (0.897)	0.231 (0.029)
AL	-0.271 (0.037)	-0.233 (0.073)	-0.033 (0.803)	-0.024 (0.855)
SFCT	0.093 (0.383)	-0.010 (0.928)	-0.003 (0.980)	-0.731 (<0.001)
CVI	-0.222 (0.035)	-0.238 (0.024)	-0.016 (0.883)	-0.275 (0.004)
TCA	0.088 (0.409)	0.008 (0.938)	0.026 (0.810)	-0.565 (<0.001)
LA	0.046 (0.669)	-0.033 (0.760)	0.017 (0.871)	-0.582 (<0.001)
SA	0.167 (0.115)	0.092 (0.389)	0.040 (0.705)	-0.477 (<0.001)
Mean choroidal reflectivity	0.190 (0.073)	0.222 (0.035)	0.043 (0.685)	
Peak reflectivity of the RPE-BM complex	0.216 (0.041)	-0.216 (0.041)	0.067 (0.528)	-0.059 (0.579)
Peak reflectivity of the OBC		0.906 (<0.001)	-0.435 (<0.001)	0.190 (0.073)
Ratio of reflectivity of the OBC to the RPE-BM complex	0.906 (<0.001)		-0.449 (<0.001)	0.222 (0.035)
Latency from the RPE-BM complex to the OBC	-0.435 (<0.001)	-0.449 (<0.001)		0.043 (0.685)

AL, Axial length; SFCT, Subfoveal choroidal thickness; CVI, Choroid vascular index; TCA, Total choroidal area; LA, Luminal area; SA, Stromal area; OBC, Optical barrier of the choroid; RPE, Retinal pigment epithelium; BM, Bruch’s membrane

^a^ Pearson’s correlation, P<0.05 was considered to be statistically significant.

### Variations in the optical barrier of the choroid in the diseased eyes

**Fig 2:**
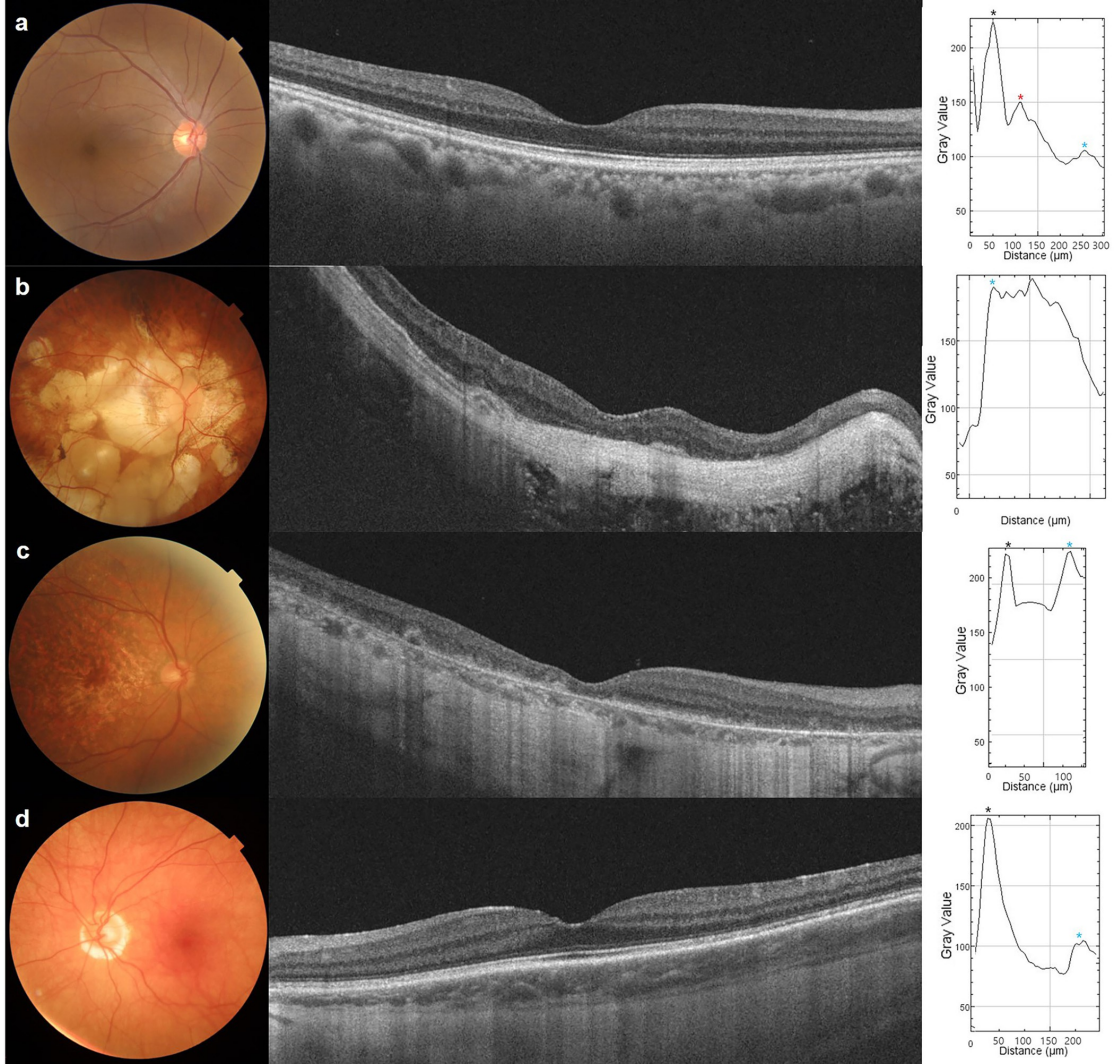
Variations in the optical barrier of the choroid in the diseased eyes. Choroidal reflectivity was measured in the eye with a normal fundus (a), pathologic myopia (b), geographic atrophy (c), and Vogt-Koyanagi-Harada disease (d). In eyes with a normal fundus, the OBC was detected on OCT and in the plot (a). Because the choroid was very thin in the eyes with pathological myopia, the OBC was not detected on OCT and in the plot (b). In the eyes with geographic atrophy and Vogt-Koyanagi-Harada disease, no peak reflectivity presented on the OBC between RPE-BM complex and chorioscleral interface reflectivity (c, d). The black asterisk indicates RPE-BM complex reflectivity, the red asterisk indicates peak reflectivity of the OBC, and the blue asterisk indicates the chorioscleral interface.

We included three eyes with chorioretinal diseases. One eye had a sunset glow fundus due to Vogt-Koyanagi-Harada disease (Fig 2D). Others had pathologic myopia (Fig 2B) or geographic atrophy involving the macular center (Fig 2C). The OBC was not detected in any eye with a sunset glow fundus due to Vogt-Koyanagi-Harada disease, pathologic myopia, or geographic atrophy.

## Discussion

The choroidal reflectance on an OCT image varies with the depth of the reflected signal because of signal decay [1]. The choroidal reflectance can also vary with the distribution of choroidal vessels and stroma. The most characteristic structure for choroidal reflectivity on OCT images is the choroidal vessel itself, and because of the low reflectivity originating from the inside of the blood vessel, the distribution of choroidal reflectivity can change depending on the distribution pattern of large blood vessels [14, 15]. In the current study, we plotted the choroidal reflectance on OCT images. Moreover, we identified the optical barrier approximately 45 μm below the RPE-BM complex layer. According to previous studies reporting the thickness of the retinal and choroidal layers in the normal eye, RPE-Bruch’s membrane complex was approximately 25.09 μm, the choriocapillaris was approximately 9.89 μm, and the choriocapillaris together with Sattler’s layer measured approximately 87.22 μm [16–18]. In a separate histological study, mean choriocapillaris thickness was measured at 6.8 ± 2.5 μm at the center [19]. The anatomical location of the optical barrier thus corresponds to the boundary between the choriocapillaris and Sattler’s layer or within Sattler’s layer. When matched with an en-face image, the layer was composed of the anterior vascular wall of the choroidal blood vessels and choroidal stroma (S1 Fig). The presence of relatively few vascular lumens at this location compared with those in the choroids at other depths may have contributed to the formation of OBCs. Recent studies have revealed that the distribution of hyperreflective choroidal foci in the choroidal stroma can contribute to changes in choroidal reflectance on en face images [7, 8]. They suggested that the foci might have originated from melanin pigment. The presence of abundant hyperreflective choroidal foci at this location might also have contributed to OBC formation. Light entering the eye penetrates the relatively transparent retina and is perceived through photoreceptors in the retina. The RPE layer absorbs light that passes through the retina through the melanin pigment and plays an important role in preventing the scattering of transmitted light [20–22]. Melanin pigments are also present in the choroid and may play an important role in preventing light scattering. The role of melanin within the choroid encompasses light absorption and protection of the retina through its antioxidant properties [23]. Although the RPE is rich in melanin and can absorb a large portion of visible light, choroidal vessels are easily observable behind the RPE in the real eye. This means that not only the RPE but also the melanin present in the choroid can contribute to limiting reflections within the eye. Consequently, various chorioretinal diseases associated with choroidal melanin, such as age-related macular degeneration and Vogt-Koyanagi-Harada disease, manifest in a decline in visual function [23]. In the current study, using OCT images, we attempted to measure reflectance from the RPE and choroid. Although the measurement of the difference between RPE and choroidal reflectance cannot represent the difference in contribution to preventing reflections of visible light between the RPE and choroid, the finding of the current study that the choroid has an optical barrier supports the suggestion that the melanin in the choroid may assist RPE in preventing light scattering.

In a few cases, the amplitude of the peak reflectivity of the OBC in the center, nasal, and inferior areas was not measured, although there were no cases in which all five areas (center, nasal, temporal, superior, and inferior) were not measured. However, the amplitude and latency between RPE-BM complex and OBC peak was different between macular center and perimacular area. In our study, the peak amplitude of the OBC was the highest in the center, and the latency between the RPE-BM complex and OBC peaks was shortest in the center. In contrast, in the nasal area, the peak amplitude of the OBC was relatively lower and the latency between the RPE-BM complex and OBC peaks was the longest. These findings suggest that the distribution of OBC varied with the retinal location. However, it is not clear why the characteristics of OBC varied in the retinal location. In this study, the peak reflectivity of the OBC was correlated with RPE-BM complex reflectivity. It means that the both reflectivity of the RPE-BM complex and OBC can depend on the difference in the incoming light intensity. In addition, in our cases, the ratio of peak reflectance between the OBC and RPE-BM complex was correlated with the mean choroidal reflectivity (Table 4). These suggest that the reflectivity of OBC depends on the variation of choroidal reflectivity itself. Miura et al. reported that the melanin occupancy rate in the choroid is significantly lower in the nasal area [23]. And the difference in the distribution of melanin might have contribute the variation of the OBC. We showed that the variation of the OBC reflectivity is related with the variation of CVI. It may suggest that the variation of choroidal reflectivity depends on the component of choroid such as vascular lumen and stroma. Our finding that axial length is related with the variation of the OBC also suggest that the choroidal change associated with axial elongation could be one of factors for variation of the OBC. In this study, we showed several cases with chorioretinal diseases. In these cases, the OBC was deteriorated. It may mean that choroidal changes in diseased eyes can be a factor for variations in the OBC. However, the study was aimed at investigating choroidal reflectivity in normal eyes. Variations in the OBC in diseased eyes require further studies with many cases for each type of chorioretinal disease.

This study had some limitations. First, it was a retrospective study. Second, the number of highly myopic eyes was relatively small compared with that of non-high myopic eyes. Therefore, it may be difficult to determine the association between high myopia and choroidal reflectivity. In eyes with choroidal changes, such as pathologic myopia, the presence of the OBC or peak OBC reflectivity may exhibit variations based on these choroidal alterations. Third, all participants were Asians with a pigmented fundus. Non-Asians and Caucasians, who have relatively less retinal pigment than Asians, may show different results. We measured the variations in reflectivity using OCT images with a central wavelength of 1,050 nm, which cannot represent all incoming light into the chorioretina. Finally, this study did not use histological samples; therefore, we could not confirm the origin of the OBC. This study focuses on examining choroidal reflectivity within OCT images. Further histological analysis is necessary to find the origin of the OBC.

In conclusion, an optical barrier is observed on OCT images of the normal choroid. Its depth depends on the location within the macula. Most barriers are located between the choriocapillaris and Sattler’s layer or within Sattler’s layer. Further study will be mandatory to evaluate the variation of the barrier in the eyes with chorioretinal disease.

## Supporting information

S1 Fig Difference of reflectivity in OCT and OCTA according to location in eye with normal axial length. Reflectivity in OCT was measured in the right eye of a healthy 33-year-old man with 23.95 mm axial length (a). The subfoveal choroidal thickness was 324 μm. Peak reflectivity of the optical barrier of the choroid (OBC) was found at 50.99 μm from RPE-BM reflectivity. Reflectivity in en-face OCTA differed according to location (d-i). Hyporeflectivity of the outer nuclear layer in the retina is shown in the b-scan image (b) and en-face OCTA image (d, marked by the orange dotted line in c). Hyperreflectivity of RPE is shown in the b-scan image (b) and en-face OCTA image (e, marked by the yellow dotted line in c). The hyperreflectivity of Bruch’s membrane is shown in the b-scan image (b) and en-face OCTA image (f, marked by the red dotted line in c). Hyporeflectivity of the choriocapillaris is shown in the b-scan image (b) and en-face OCTA image (g, marked by the blue dotted line in c). Hyperreflectivity of the OBC is shown in the b-scan image (b) and en-face OCTA image (h, marked by the green dotted line in c). Hyporeflectivity of Haller’s layer in the choroid is shown in the b-scan image (b) and en face OCTA image (i, marked by the white dotted line in c).
(TIF)
