# Supplementary figures and images for: Quantification of choroidal hyperreflective layer: A swept-source optical coherence tomography study
Source: PLoS One. 2023 Nov 29;18(11):e0294476. doi: 10.1371/journal.pone.0294476 (PMC10686441; doi:10.1371/journal.pone.0294476)

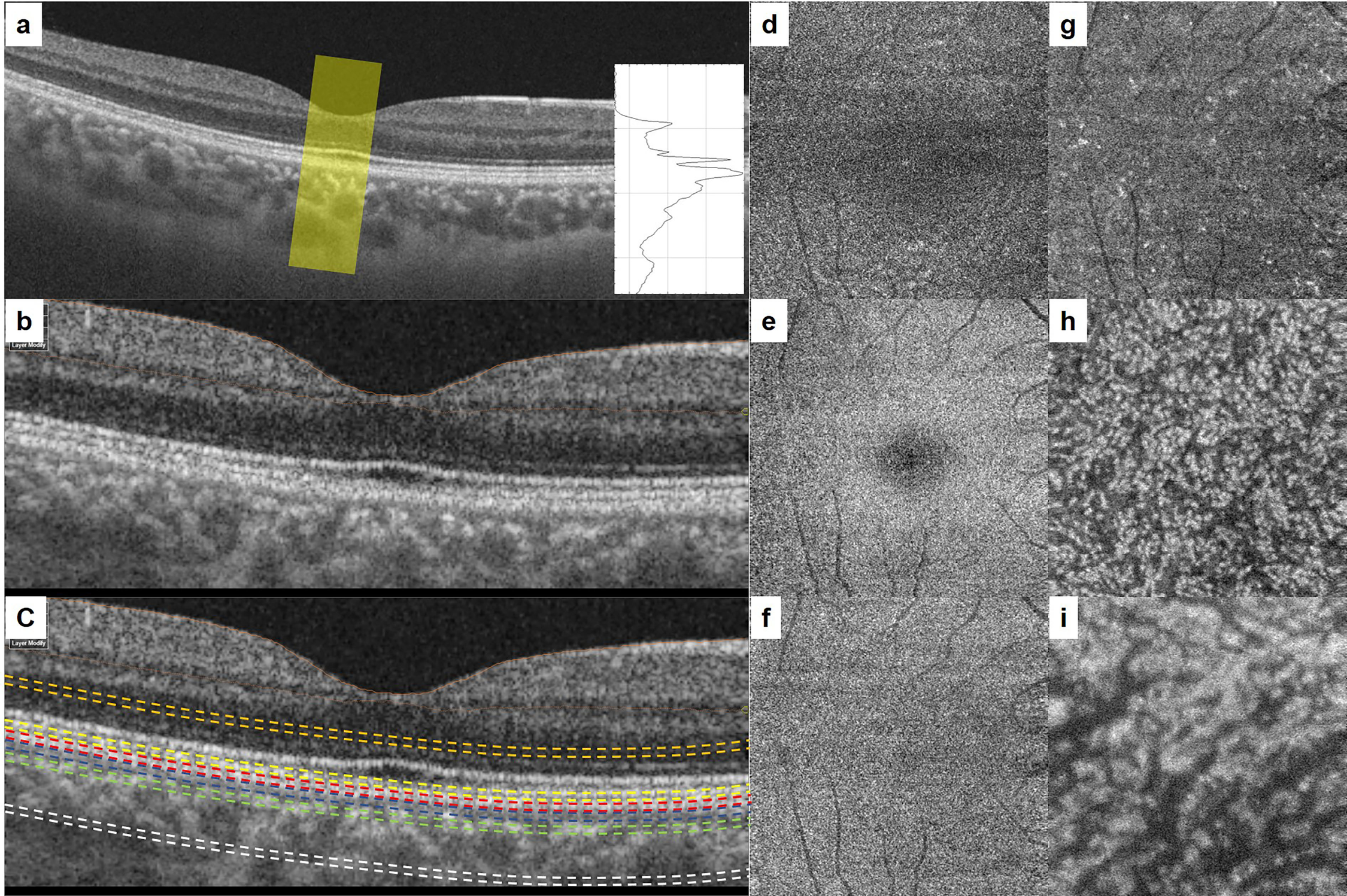

Supplement: S1 Fig — Reflectivity in OCT was measured in the right eye of a healthy 33-year-old man with 23.95 mm axial length (a). The subfoveal choroidal thickness was 324 μm. Peak reflectivity of the optical barrier of the choroid (OBC) was found at 50.99 μm from RPE-BM reflectivity. Reflectivity in en-face OCTA differed according to location (d-i). Hyporeflectivity of the outer nuclear layer in the retina is shown in the b-scan image (b) and en-face OCTA image (d, marked by the orange dotted line in c). Hyperreflectivity of RPE is shown in the b-scan image (b) and en-face OCTA image (e, marked by the yellow dotted line in c). The hyperreflectivity of Bruch’s membrane is shown in the b-scan image (b) and en-face OCTA image (f, marked by the red dotted line in c). Hyporeflectivity of the choriocapillaris is shown in the b-scan image (b) and en-face OCTA image (g, marked by the blue dotted line in c). Hyperreflectivity of the OBC is shown in the b-scan image (b) and en-face OCTA image (h, marked by the green dotted line in c). Hyporeflectivity of Haller’s layer in the choroid is shown in the b-scan image (b) and en face OCTA image (i, marked by the white dotted line in c). (TIF) [file pone.0294476.s001.tif]
